# (*Z*)-3-(2,4-Dichloro­benz­yl)-1,5-benzo­thia­zepin-4(5*H*)-one

**DOI:** 10.1107/S1600536813007435

**Published:** 2013-03-23

**Authors:** S. Murugavel, N. Manikandan, R. Selvakumar, M. Bakthadoss

**Affiliations:** a Department of Physics, Thanthai Periyar Government Institute of Technology, Vellore 632 002, India; b Department of Physics, Bharathidasan Engineering College, Nattrampalli, Vellore 635 854, India; c Department of Organic Chemistry, University of Madras, Maraimalai Campus, Chennai 600 025, India

## Abstract

In the title compound, C_16_H_11_Cl_2_NOS, the seven-membered thia­zepine ring adopts a distorted twist-boat conformation. The dihedral angle between the mean plane of the benzothia­zepine ring system and the benzene ring is 78.6 (1)°. The mol­ecular conformation is stabilized by a weak intra­molecular C—H⋯Cl hydrogen bond, which generates an *S*(5) ring motif. In the crystal, pairs of N—H⋯O hydrogen bonds link inversion-related mol­ecules into dimers, generating *R*_2_^2^(8) ring motifs. The crystal packing also features alternating π–π inter­actions between benzothia­zepine benzene rings [inter-centroid distance = 3.740 (3) Å] and dichloro­benzene rings [inter-centroid distance = 3.882 (3) Å] to consolidate a three-dimensional architecture.

## Related literature

For background to the biology and related structures of thia­zepin derivatives, see: Bakthadoss *et al.* (2013 ▶). For ring-puckering parameters, see: Cremer & Pople (1975 ▶). For hydrogen-bond motifs, see: Bernstein *et al.* (1995 ▶).
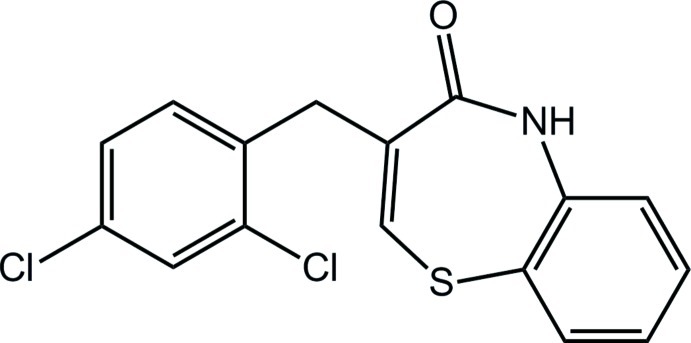


## Experimental

### 

#### Crystal data

C_16_H_11_Cl_2_NOS
*M* *_r_* = 336.22
Triclinic, 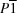

*a* = 7.879 (5) Å
*b* = 9.667 (5) Å
*c* = 9.979 (5) Å
α = 89.052 (5)°
β = 78.161 (4)°
γ = 83.647 (5)°
*V* = 739.3 (7) Å^3^
*Z* = 2
Mo *K*α radiation
μ = 0.58 mm^−1^
*T* = 293 K
0.24 × 0.21 × 0.15 mm

#### Data collection

Bruker APEXII CCD diffractometer
Absorption correction: multi-scan (*SADABS*; Sheldrick, 1996 ▶) *T*_min_ = 0.871, *T*_max_ = 0.917
18415 measured reflections
5225 independent reflections
4013 reflections with *I* > 2σ(*I*)
*R*_int_ = 0.026

#### Refinement

*R*[*F* ^2^ > 2σ(*F* ^2^)] = 0.042
*wR*(*F* ^2^) = 0.123
*S* = 1.04
5225 reflections
190 parameters
H-atom parameters constrained
Δρ_max_ = 0.55 e Å^−3^
Δρ_min_ = −0.44 e Å^−3^

### 

Data collection: *APEX2* (Bruker, 2004 ▶); cell refinement: *APEX2* and *SAINT* (Bruker, 2004 ▶); data reduction: *SAINT* and *XPREP* (Bruker, 2004 ▶); program(s) used to solve structure: *SHELXS97* (Sheldrick, 2008 ▶); program(s) used to refine structure: *SHELXL97* (Sheldrick, 2008 ▶); molecular graphics: *ORTEP-3 for Windows* (Farrugia, 2012 ▶); software used to prepare material for publication: *SHELXL97* and *PLATON* (Spek, 2009 ▶).

## Supplementary Material

Crystal structure: contains datablock(s) global, I. DOI: 10.1107/S1600536813007435/tk5208sup1.cif

Structure factors: contains datablock(s) I. DOI: 10.1107/S1600536813007435/tk5208Isup2.hkl

Supplementary material file. DOI: 10.1107/S1600536813007435/tk5208Isup3.cml

Additional supplementary materials:  crystallographic information; 3D view; checkCIF report

## Figures and Tables

**Table 1:** Hydrogen-bond geometry (Å, °)

*D*—H⋯*A*	*D*—H	H⋯*A*	*D*⋯*A*	*D*—H⋯*A*
C10—H10*B*⋯Cl1	0.97	2.64	3.103 (3)	109
N1—H1*A*⋯O1^i^	0.86	2.10	2.873 (2)	149

Symmetry code: (i) 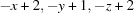.

**Fig. 1.**
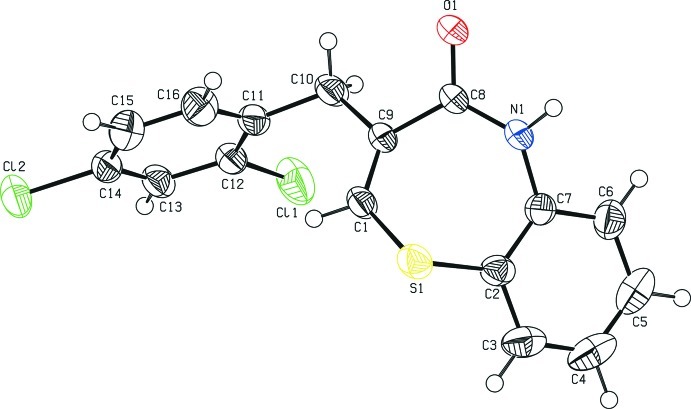
Molecular structure of the title compound showing displacement ellipsoids at the 30% probability level. H atoms are presented as a small spheres of arbitrary radii.

**Fig. 2.**
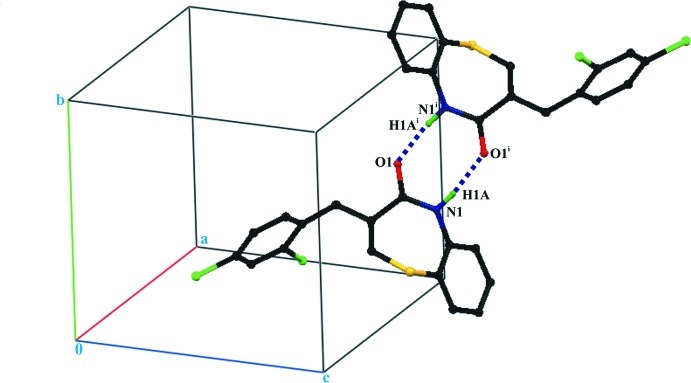
Part of the crystal structure of the title compound showing N—H···O intermolecular hydrogen bonds (dotted lines) generating R22(8) centrosymmetric dimers [Symmetry code: (i) 2-x, 1-y, 2-z].

**Fig. 3.**
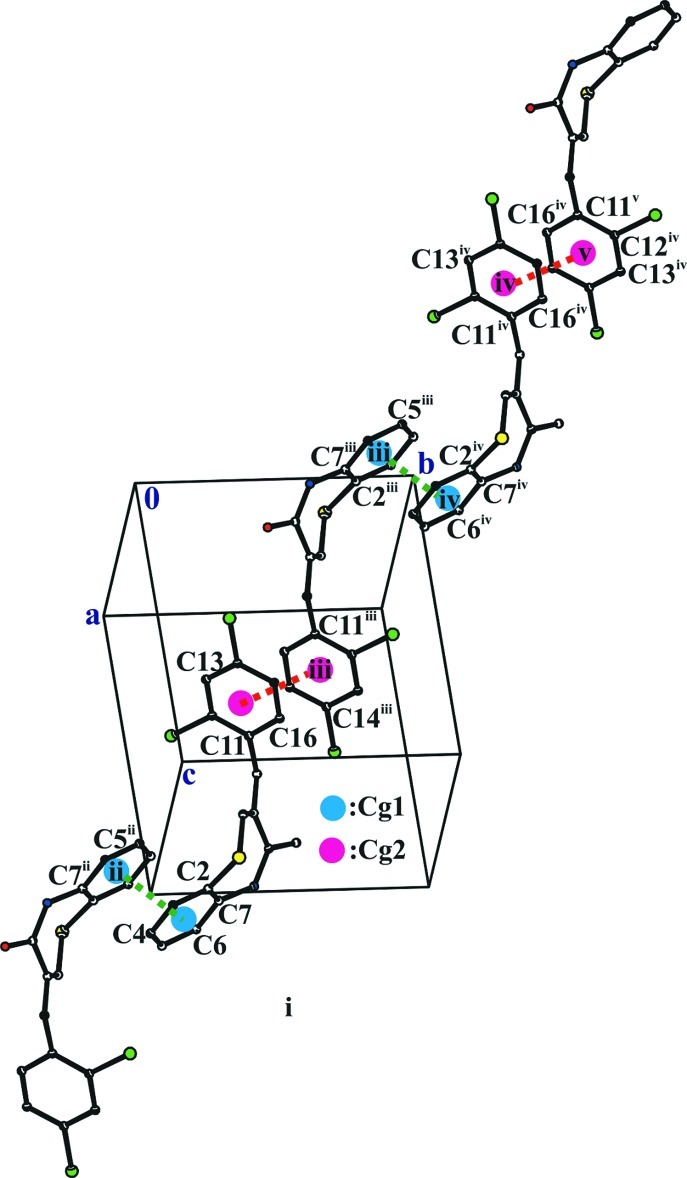
A view of alternating π—π interactions forming supramolecular stacks along the a axis in the crystal structure of the title compound. Cg1 and Cg2 are the centroids of the (C2–C7) and (C11–C16) benzene rings, respectively [Symmetry code: (ii) 2-x, -y, 2-z; (iii) 1-x, 1-y, 1-z; (iv) -1+x, 1+y, -1+z; (v) -x, 2-y, -z].

## supplementary crystallographic information

### Comment

The background to the biology and related structures of thiazepin derivatives, has been described recently (Bakthadoss *et al.*, 2013). In view of this biological importance, the crystal structure of the title compound has been carried out and the results are presented here.

Fig. 1. shows a displacement ellipsoid plot of (I), with the atom numbering scheme. The seven membered thiazepine ring (N1/S1/C1/C2/C7/C8/C9) adopts distorted twist-boat conformation as indicated by puckering parameters (Cremer & Pople, 1975): QT = 0.8962 (12) Å, φ_2_ = 353.2 (1)° and φ_3_ = 358.9 (3)°. The atom O1 deviates by 0.667 (1) Å from the least-squares plane of the thiazepin ring. The dihedral angle between the benzothiazepin ring system and the benzene ring is 78.6 (1)°. The atoms Cl1 and Cl2 deviate by 0.075 (1) and -0.006 (1) Å, respectively, from the plane of the attached benzene ring (C11–C16). The sum of angles at N1 atom of the thiazepin ring (360.0°) is in accordance with *sp*^2^ hybridization. The geometric parameters of the title molecule agree well with those reported for similar structures (Bakthadoss *et al.*, 2013).

The molecular conformation is stabilized by a weak intramolecular C10—H10B···Cl1 hydrogen bond, which generates an *S*(5) ring motif (Bernstein *et al.*, 1995). In the crystal packing, molecules are linked by N1—H1A···O1 hydrogen bonds into cyclic centrosymmetric *R_2_^2^*(8) dimers (Fig. 2 and Table 1). The crystal packing is further stabilized by alternating π–π interactions with *Cg*1···*Cg*1^ii^ = 3.740 (3) Å (symmetry code: (ii) = 2*-x, -y,* 2*-z*) and *Cg*2···*Cg*2^iii^ = 3.882 (3) Å (symmetry code: (iii) = 1*-x,* 1*-y,* 1*-z*) forming supramolecular stacks along the *a* axis (Fig. 3; *Cg*1 and *Cg*2 are the centroids of the C2–C7 and C11–C16 benzene rings, respectively).

### Experimental

A mixture of (*Z*)-methyl 2-(bromomethyl)-3-(2,4-dichlorophenyl)acrylate 2 mmol) and *o*-aminothiophenol (2 mmol) in the presence of potassium *tert*-butoxide (4.8 mmol) in dry THF (10 ml) was stirred at room temperature for 1 h. After the completion of the reaction as indicated by TLC, the reaction mixture was concentrated and the resulting crude mass was diluted with water (20 ml) and extracted with ethyl acetate (3 *x* 20 ml). The organic layer was washed with brine (2 *x* 20 ml) and dried over anhydrous sodium sulfate. The organic layer was concentrated, which successfully provide the crude final product ((*Z*)-3-(2,4dichlorobenzyl)benzo[*b*][1,4]thiazepin-4(5*H*)-one). The final product was purified by column chromatography on silica gel to afford the title compound in good yield (42%). Single crystals suitable for X-ray diffraction were obtained by slow evaporation of its ethylacetate solution at room temperature.

### Refinement

All the H atoms were positioned geometrically and constrained to ride on their parent atom with C—H = 0.93–0.97 Å and N—H = 0.86 Å, and with *U*_iso_(H)=1.5*U*_eq_ for methyl H atoms and 1.2*U*_eq_(C) for other H atoms.

### Crystal data

**Table:**

C_16_H_11_Cl_2_NOS	*Z* = 2
*M**_r_* = 336.22	*F*(000) = 344
Triclinic, *P*1	*D*_x_ = 1.510 Mg m^−^^3^
Hall symbol: -P 1	Mo *K*α radiation, λ = 0.71073 Å
*a* = 7.879 (5) Å	Cell parameters from 5914 reflections
*b* = 9.667 (5) Å	θ = 2.1–33.7°
*c* = 9.979 (5) Å	µ = 0.58 mm^−^^1^
α = 89.052 (5)°	*T* = 293 K
β = 78.161 (4)°	Block, colourless
γ = 83.647 (5)°	0.24 × 0.21 × 0.15 mm
*V* = 739.3 (7) Å^3^	

### Data collection

**Table:**

Bruker APEXII CCD diffractometer	5225 independent reflections
Radiation source: fine-focus sealed tube	4013 reflections with *I* > 2σ(*I*)
Graphite monochromator	*R*_int_ = 0.026
Detector resolution: 10.0 pixels mm^-1^	θ_max_ = 33.7°, θ_min_ = 2.1°
ω scans	*h* = −12→11
Absorption correction: multi-scan (*SADABS*; Sheldrick, 1996)	*k* = −14→14
*T*_min_ = 0.871, *T*_max_ = 0.917	*l* = −14→15
18415 measured reflections	

### Refinement

**Table:**

Refinement on *F*^2^	Primary atom site location: structure-invariant direct methods
Least-squares matrix: full	Secondary atom site location: difference Fourier map
*R*[*F*^2^ > 2σ(*F*^2^)] = 0.042	Hydrogen site location: inferred from neighbouring sites
*wR*(*F*^2^) = 0.123	H-atom parameters constrained
*S* = 1.04	*w* = 1/[σ^2^(*F*_o_^2^) + (0.0583*P*)^2^ + 0.2157*P*] where *P* = (*F*_o_^2^ + 2*F*_c_^2^)/3
5225 reflections	(Δ/σ)_max_ = 0.001
190 parameters	Δρ_max_ = 0.55 e Å^−^^3^
0 restraints	Δρ_min_ = −0.44 e Å^−^^3^

### Special details

**Table:**

Geometry. All e.s.d.'s (except the e.s.d. in the dihedral angle between two l.s. planes) are estimated using the full covariance matrix. The cell e.s.d.'s are taken into account individually in the estimation of e.s.d.'s in distances, angles and torsion angles; correlations between e.s.d.'s in cell parameters are only used when they are defined by crystal symmetry. An approximate (isotropic) treatment of cell e.s.d.'s is used for estimating e.s.d.'s involving l.s. planes.
Refinement. Refinement of *F*^2^ against ALL reflections. The weighted *R*-factor *wR* and goodness of fit *S* are based on *F*^2^, conventional *R*-factors *R* are based on *F*, with *F* set to zero for negative *F*^2^. The threshold expression of *F*^2^ > σ(*F*^2^) is used only for calculating *R*-factors(gt) *etc*. and is not relevant to the choice of reflections for refinement. *R*-factors based on *F*^2^ are statistically about twice as large as those based on *F*, and *R*- factors based on ALL data will be even larger.

### Fractional atomic coordinates and isotropic or equivalent isotropic displacement parameters (Å^2^)

**Table:**

	*x*	*y*	*z*	*U*_iso_*/*U*_eq_	
C1	0.53450 (18)	0.28937 (15)	0.94205 (15)	0.0366 (3)	
H1	0.4546	0.2673	0.8911	0.044*	
C2	0.6952 (2)	0.14986 (15)	1.12880 (14)	0.0371 (3)	
C3	0.6971 (3)	0.01550 (17)	1.18247 (17)	0.0510 (4)	
H3	0.5951	−0.0277	1.1996	0.061*	
C4	0.8473 (3)	−0.05324 (19)	1.21016 (19)	0.0622 (5)	
H4	0.8466	−0.1424	1.2467	0.075*	
C5	0.9993 (3)	0.0092 (2)	1.18406 (19)	0.0600 (5)	
H5	1.1010	−0.0374	1.2038	0.072*	
C6	1.0008 (2)	0.14161 (19)	1.12835 (17)	0.0469 (3)	
H6	1.1037	0.1836	1.1106	0.056*	
C7	0.84959 (19)	0.21138 (14)	1.09904 (14)	0.0352 (3)	
C8	0.79730 (18)	0.41136 (14)	0.94238 (15)	0.0349 (3)	
C9	0.66727 (17)	0.35381 (13)	0.87569 (14)	0.0321 (2)	
C10	0.6884 (2)	0.39317 (17)	0.72620 (15)	0.0423 (3)	
H10A	0.6968	0.4924	0.7186	0.051*	
H10B	0.7978	0.3460	0.6767	0.051*	
C11	0.5449 (2)	0.35964 (15)	0.65712 (14)	0.0371 (3)	
C12	0.55727 (19)	0.24270 (15)	0.57559 (15)	0.0359 (3)	
C13	0.42879 (19)	0.21821 (15)	0.50589 (15)	0.0388 (3)	
H13	0.4419	0.1402	0.4499	0.047*	
C14	0.28122 (19)	0.31163 (16)	0.52118 (14)	0.0390 (3)	
C15	0.2600 (2)	0.42737 (18)	0.60401 (18)	0.0477 (4)	
H15	0.1583	0.4886	0.6158	0.057*	
C16	0.3933 (2)	0.45071 (17)	0.66928 (17)	0.0469 (4)	
H16	0.3808	0.5302	0.7232	0.056*	
N1	0.85975 (16)	0.34776 (12)	1.04633 (13)	0.0375 (3)	
H1A	0.9155	0.3990	1.0879	0.045*	
O1	0.84965 (17)	0.52292 (12)	0.89978 (14)	0.0545 (3)	
Cl1	0.73749 (6)	0.11906 (5)	0.55837 (6)	0.06427 (15)	
Cl2	0.11969 (6)	0.27905 (6)	0.43563 (5)	0.05837 (14)	
S1	0.49323 (5)	0.24136 (4)	1.11466 (4)	0.04389 (11)	

### Atomic displacement parameters (Å^2^)

**Table:**

	*U*^11^	*U*^22^	*U*^33^	*U*^12^	*U*^13^	*U*^23^
C1	0.0354 (7)	0.0435 (7)	0.0355 (7)	−0.0151 (5)	−0.0122 (5)	0.0003 (5)
C2	0.0443 (7)	0.0377 (6)	0.0304 (6)	−0.0118 (5)	−0.0059 (5)	0.0013 (5)
C3	0.0692 (11)	0.0425 (8)	0.0405 (8)	−0.0178 (8)	−0.0032 (7)	0.0067 (6)
C4	0.0933 (16)	0.0444 (9)	0.0444 (9)	−0.0023 (9)	−0.0077 (9)	0.0139 (7)
C5	0.0720 (13)	0.0603 (11)	0.0463 (9)	0.0113 (9)	−0.0196 (9)	0.0086 (8)
C6	0.0460 (8)	0.0559 (9)	0.0417 (8)	−0.0047 (7)	−0.0169 (7)	0.0046 (7)
C7	0.0413 (7)	0.0372 (6)	0.0298 (6)	−0.0090 (5)	−0.0111 (5)	0.0016 (5)
C8	0.0351 (6)	0.0342 (6)	0.0405 (7)	−0.0127 (5)	−0.0153 (5)	0.0023 (5)
C9	0.0344 (6)	0.0322 (6)	0.0339 (6)	−0.0106 (5)	−0.0129 (5)	−0.0001 (5)
C10	0.0475 (8)	0.0506 (8)	0.0355 (7)	−0.0246 (7)	−0.0142 (6)	0.0037 (6)
C11	0.0453 (7)	0.0411 (7)	0.0295 (6)	−0.0162 (6)	−0.0121 (5)	0.0028 (5)
C12	0.0359 (7)	0.0376 (6)	0.0369 (7)	−0.0105 (5)	−0.0103 (5)	0.0015 (5)
C13	0.0416 (7)	0.0419 (7)	0.0367 (7)	−0.0144 (6)	−0.0114 (6)	−0.0024 (5)
C14	0.0392 (7)	0.0492 (8)	0.0328 (6)	−0.0151 (6)	−0.0121 (5)	0.0086 (5)
C15	0.0457 (8)	0.0507 (9)	0.0466 (8)	0.0005 (7)	−0.0122 (7)	−0.0001 (7)
C16	0.0563 (10)	0.0449 (8)	0.0416 (8)	−0.0057 (7)	−0.0144 (7)	−0.0072 (6)
N1	0.0406 (6)	0.0388 (6)	0.0405 (6)	−0.0169 (5)	−0.0194 (5)	0.0046 (5)
O1	0.0638 (8)	0.0471 (6)	0.0703 (8)	−0.0323 (6)	−0.0422 (6)	0.0212 (5)
Cl1	0.0451 (2)	0.0557 (3)	0.0965 (4)	0.00095 (18)	−0.0269 (2)	−0.0159 (2)
Cl2	0.0465 (2)	0.0776 (3)	0.0608 (3)	−0.0192 (2)	−0.0275 (2)	0.0075 (2)
S1	0.03570 (19)	0.0574 (2)	0.0392 (2)	−0.01553 (16)	−0.00402 (14)	0.00582 (15)

### Geometric parameters (Å, º)

**Table:**

C1—C9	1.3294 (19)	C8—C9	1.4901 (18)
C1—S1	1.7508 (17)	C9—C10	1.514 (2)
C1—H1	0.9300	C10—C11	1.505 (2)
C2—C7	1.388 (2)	C10—H10A	0.9700
C2—C3	1.396 (2)	C10—H10B	0.9700
C2—S1	1.7642 (18)	C11—C12	1.387 (2)
C3—C4	1.368 (3)	C11—C16	1.389 (2)
C3—H3	0.9300	C12—C13	1.383 (2)
C4—C5	1.376 (3)	C12—Cl1	1.7336 (17)
C4—H4	0.9300	C13—C14	1.375 (2)
C5—C6	1.387 (3)	C13—H13	0.9300
C5—H5	0.9300	C14—C15	1.377 (2)
C6—C7	1.385 (2)	C14—Cl2	1.7295 (16)
C6—H6	0.9300	C15—C16	1.384 (2)
C7—N1	1.4158 (19)	C15—H15	0.9300
C8—O1	1.2350 (17)	C16—H16	0.9300
C8—N1	1.3481 (19)	N1—H1A	0.8600
			
C9—C1—S1	127.26 (11)	C11—C10—H10A	108.3
C9—C1—H1	116.4	C9—C10—H10A	108.3
S1—C1—H1	116.4	C11—C10—H10B	108.3
C7—C2—C3	119.11 (15)	C9—C10—H10B	108.3
C7—C2—S1	121.99 (12)	H10A—C10—H10B	107.4
C3—C2—S1	118.67 (13)	C12—C11—C16	116.63 (14)
C4—C3—C2	120.73 (17)	C12—C11—C10	123.35 (15)
C4—C3—H3	119.6	C16—C11—C10	119.97 (14)
C2—C3—H3	119.6	C13—C12—C11	122.51 (14)
C3—C4—C5	120.14 (17)	C13—C12—Cl1	117.07 (12)
C3—C4—H4	119.9	C11—C12—Cl1	120.42 (12)
C5—C4—H4	119.9	C14—C13—C12	118.59 (14)
C4—C5—C6	119.97 (18)	C14—C13—H13	120.7
C4—C5—H5	120.0	C12—C13—H13	120.7
C6—C5—H5	120.0	C13—C14—C15	121.28 (14)
C7—C6—C5	120.20 (17)	C13—C14—Cl2	118.34 (12)
C7—C6—H6	119.9	C15—C14—Cl2	120.38 (13)
C5—C6—H6	119.9	C14—C15—C16	118.61 (16)
C6—C7—C2	119.78 (14)	C14—C15—H15	120.7
C6—C7—N1	117.04 (13)	C16—C15—H15	120.7
C2—C7—N1	123.08 (13)	C15—C16—C11	122.33 (15)
O1—C8—N1	118.83 (12)	C15—C16—H16	118.8
O1—C8—C9	117.93 (12)	C11—C16—H16	118.8
N1—C8—C9	123.23 (12)	C8—N1—C7	131.25 (11)
C1—C9—C8	124.33 (13)	C8—N1—H1A	114.4
C1—C9—C10	122.86 (12)	C7—N1—H1A	114.4
C8—C9—C10	112.36 (11)	C1—S1—C2	101.45 (7)
C11—C10—C9	115.75 (12)		
			
C7—C2—C3—C4	2.3 (2)	C16—C11—C12—C13	1.8 (2)
S1—C2—C3—C4	−172.38 (14)	C10—C11—C12—C13	−175.75 (13)
C2—C3—C4—C5	−0.6 (3)	C16—C11—C12—Cl1	−177.66 (12)
C3—C4—C5—C6	−0.6 (3)	C10—C11—C12—Cl1	4.7 (2)
C4—C5—C6—C7	0.1 (3)	C11—C12—C13—C14	−1.8 (2)
C5—C6—C7—C2	1.6 (2)	Cl1—C12—C13—C14	177.71 (11)
C5—C6—C7—N1	178.10 (15)	C12—C13—C14—C15	−0.1 (2)
C3—C2—C7—C6	−2.8 (2)	C12—C13—C14—Cl2	−179.09 (11)
S1—C2—C7—C6	171.71 (12)	C13—C14—C15—C16	1.9 (2)
C3—C2—C7—N1	−179.04 (14)	Cl2—C14—C15—C16	−179.17 (13)
S1—C2—C7—N1	−4.5 (2)	C14—C15—C16—C11	−1.8 (3)
S1—C1—C9—C8	−7.0 (2)	C12—C11—C16—C15	0.0 (2)
S1—C1—C9—C10	−178.71 (12)	C10—C11—C16—C15	177.70 (15)
O1—C8—C9—C1	−141.76 (16)	O1—C8—N1—C7	−167.01 (16)
N1—C8—C9—C1	38.2 (2)	C9—C8—N1—C7	13.1 (2)
O1—C8—C9—C10	30.69 (19)	C6—C7—N1—C8	135.73 (17)
N1—C8—C9—C10	−149.37 (15)	C2—C7—N1—C8	−47.9 (2)
C1—C9—C10—C11	2.2 (2)	C9—C1—S1—C2	−51.65 (16)
C8—C9—C10—C11	−170.34 (13)	C7—C2—S1—C1	58.57 (13)
C9—C10—C11—C12	−98.99 (18)	C3—C2—S1—C1	−126.89 (13)
C9—C10—C11—C16	83.49 (19)		

### Hydrogen-bond geometry (Å, º)

**Table:**

*D*—H···*A*	*D*—H	H···*A*	*D*···*A*	*D*—H···*A*
C10—H10*B*···Cl1	0.97	2.64	3.103 (3)	109
N1—H1*A*···O1^i^	0.86	2.10	2.873 (2)	149

Symmetry code: (i) −*x*+2, −*y*+1, −*z*+2.
